# Activation of the GPR35 pathway drives angiogenesis in the tumour microenvironment

**DOI:** 10.1136/gutjnl-2020-323363

**Published:** 2021-03-23

**Authors:** Ester Pagano, Joshua E Elias, Georg Schneditz, Svetlana Saveljeva, Lorraine M Holland, Francesca Borrelli, Tom H Karlsen, Arthur Kaser, Nicole C Kaneider

**Affiliations:** 1 Cambridge Institute of Therapeutic Immunology & Infectious Disease (CITIID), Jeffrey Cheah Biomedical Centre, Cambridge Biomedical Campus, University of Cambridge, Cambridge, UK; 2 Department of Pharmacy, School of Medicine and Surgery, University of Naples Federico II, Naples, Italy; 3 Division of Gastroenterology and Hepatology, Department of Medicine, Addenbrooke's Hospital, University of Cambridge, Cambridge, UK; 4 Norwegian PSC Research Center, Oslo University Hospital and Institute of Clinical Medicine, University of Oslo, Oslo, Norway

**Keywords:** colorectal cancer, primary sclerosing cholangitis, angiogenesis, ulcerative colitis, receptor characterisation

## Abstract

**Objective:**

Primary sclerosing cholangitis (PSC) is in 70% of cases associated with inflammatory bowel disease. The hypermorphic T108M variant of the orphan G protein-coupled receptor GPR35 increases risk for PSC and ulcerative colitis (UC), conditions strongly predisposing for inflammation-associated liver and colon cancer. Lack of GPR35 reduces tumour numbers in mouse models of spontaneous and colitis associated cancer. The tumour microenvironment substantially determines tumour growth, and tumour-associated macrophages are crucial for neovascularisation. We aim to understand the role of the GPR35 pathway in the tumour microenvironment of spontaneous and colitis-associated colon cancers.

**Design:**

Mice lacking GPR35 on their macrophages underwent models of spontaneous colon cancer or colitis-associated cancer. The role of tumour-associated macrophages was then assessed in biochemical and functional assays.

**Results:**

Here, we show that GPR35 on macrophages is a potent amplifier of tumour growth by stimulating neoangiogenesis and tumour tissue remodelling. Deletion of *Gpr35* in macrophages profoundly reduces tumour growth in inflammation-associated and spontaneous tumour models caused by mutant tumour suppressor adenomatous polyposis coli. Neoangiogenesis and matrix metalloproteinase activity is promoted by GPR35 via Na/K-ATPase-dependent ion pumping and Src activation, and is selectively inhibited by a GPR35-specific pepducin. Supernatants from human inducible-pluripotent-stem-cell derived macrophages carrying the UC and PSC risk variant stimulate tube formation by enhancing the release of angiogenic factors.

**Conclusions:**

Activation of the GPR35 pathway promotes tumour growth via two separate routes, by directly augmenting proliferation in epithelial cells that express the receptor, and by coordinating macrophages’ ability to create a tumour-permissive environment.

**Significance of this study**

**What is already known on this subject?**

- A coding variant (T108M) in the G protein-coupled receptor GPR35 increases the risk for primary sclerosing cholangitis (PSC) and ulcerative colitis (UC). PSC and UC are chronic inflammatory conditions that significantly increase the risk for the development of cancer, and GPR35 is highly expressed in colorectal cancer.
- GPR35 interacts with the sodium-potassium pump (Na/K-ATPase). Na/K-ATPase is the quintessential controller of a cell’s electrochemical gradient and controls Src-kinase signalling. The UC/PSC-associated GPR35^T108M^ is hypermorphic and promotes the pump’s ion exchange and signal transduction function resulting in increased proliferation of intestinal epithelial cells. Mice lacking Gpr35 globally or conditionally in their Vln^+^ cells (intestinal epithelial cells) develop reduced numbers of tumours in sporadic cancer and colitis-associated cancer.

**Significance of this study**

**What are the new findings?**

- Tumours lacking GPR35 are smaller compared with wild-type tumours. Deletion of *Gpr35* in LysM^+^ cells reduces tumour growth substantially. GPR35 on macrophages increases the secretion of the angiogenic factors CXCL-1 (the murine orthologue of interleukin 8 (IL-8)) and VEGF. GPR35 on LysM^+^ cells promotes vessel sprouting from ex vivo cultured aortic rings which is dependent on its interaction with the Na/K-ATPase. Loss of GPR35 on macrophages reduces matrix metalloproteinase activity in tumour tissue, a prerequisite for tissue remodelling and angiogenesis. Macrophages carrying the human risk variant GPR35^T108M^ secrete substantially more VEGF, IL-8 and matrix-metallo-proteinases (MMP2). Risk variant supernatants induce tube formation in human microvascular endothelial cells. Pharmacological pepducin-based GPR35 blockade markedly reduces tumour size and tissue levels of CXCL-1, VEGF, MMP2 and MMP9. GPR35 in macrophages is an upstream central coordinator of tissue remodelling and tumour-promotion, creating a tumour-permissive environment.

**How might it impact on clinical practice in the foreseeable future?**

- Anti-VEGF antibodies are approved in cancer therapy to reduce tumour growth and angiogenesis, anti-IL-8 antibodies are in phase 2 clinical trials and have huge promise to slow down tumour angiogenesis.
- Blocking GPR35 could be very promising in cancer therapy as this reduces the release of both, VEGF and IL-8 and reduces tumour cell proliferation by inhibiting neoangiogenesis.

## Introduction

Colorectal cancer (CRC) is the leading cause of death from gastrointestinal malignancy, and the second most common cause of cancer-related death in the western world.^1^ Mutations in the adenomatous polyposis coli (*APC*) gene are a key event in both spontaneous and hereditary CRC.^2^ A subset of CRC is due to intestinal inflammation, referred to as colitis-associated cancer (CAC).^3^ CAC typically arises from long-standing ulcerative colitis (UC), and is particularly frequent in a variant of UC (often referred to as PSC-inflammatory bowel disease (IBD)) that is often present in patients with primary sclerosing cholangitis (PSC). PSC and UC are chronic inflammatory conditions of the bile ducts and the large intestine, respectively.^4 5^ Both diseases have an extreme cumulative risk for the development of cancer.^6 7^

A coding variant in *GPR35* (rs3749171, leading to a T108M substitution) is associated with risk for PSC and UC.^8 9^ GPR35 is an orphan G protein-coupled receptor that interacts with the sodium-potassium pump (Na/K-ATPase).^10^ Na/K-ATPase is the quintessential controller of a cell’s electrochemical gradient, for which ~30% of cellular energy in the form of ATP is expended. The GPR35 interaction promotes Na/K-ATPase’s pump function, secondarily controlling Ca^2+^ homoeostasis. The UC/PSC-associated GPR35^T108M^ variant is hypermorphic in its stimulatory activity on the pump.^10^ Na/K-ATPase acts as a scaffold for the non-receptor tyrosine kinase Src, and modulates its phosphorylation.^11^ GPR35, via its Na/K-ATPase interaction, regulates the proliferation of intestinal epithelial cells by phosphorylation and activation of Src.^10^ Indeed mice lacking *Gpr35* develop fewer tumours in a sporadic murine cancer model (in mice carrying an *Apc^min^* allele) and in a model of CAC induced by the mutagen azoxymethane followed by dextran sodium sulphate (AOM-DSS).^10^ GRP35 is known to be highly expressed in human CRC^12 13^ and deletion of *Gpr35* selectively in the intestinal epithelium was sufficient to reduce tumour numbers.^10^ Kaya *et al* report that LPA activation of GPR35 in CX3CR1+macrophages maintains tumour necrosis factor-mediated intestinal homoeostasis and thereby reduces acute inflammation.^14^

Mutations in oncogenes and tumour-suppressor genes lead to unorganised proliferation and reduced cell death which initiates the development of malignancies. Tumour growth is not only determined by signal transduction pathways leading to proliferation,^15^ but also by neoangiogenesis.^16^ The tumour microenvironment (TME) consists of several cell types including leukocytes,^17 18^ as well as the extracellular matrix, which are all important for growth and progression of tumours.^19^ Tumour cells themselves have the capability to recruit cells of the TME by releasing chemokines, cytokines and proteinases.^20^ This cooperation between cells of the TME and malignant cells leads to tissue remodelling and angiogenesis within the tumour, resulting in enhanced proliferation and metastatic capability.^21^

Atypical angiogenesis does not only play an important role in tumour neo-vascularisation,^22^ but is also a hallmark of chronic inflammation including IBD.^23^ Release of CXCL-1 (KC, GROα) and/or CXCL-8 (interleukin 8, IL-8) together with VEGF initiate tumour angiogenesis and lead to tumour growth by guaranteeing sufficient supply of nutrients.^24^ These cytokines are also increased in IBD and augment neovascularisation in areas of tissue regeneration.^23^ Tumour-associated macrophages (TAMs) as part of the TME can resemble either proinflammatory M1 macrophages or M2-like macrophages with immunosuppressive and tumour-promoting properties.^18 25^ Increased numbers of TAMs, in particular M2-like cells, directly correlate with poor prognosis of cancer by stimulating angiogenesis, modulating tumour cell invasion, cell motility as well as persistent growth.^26–28^ TAMs also express proteinases including matrix metalloproteinases (MMPs), which play a key role in angiogenesis and progression of tumours.^29^ Neutrophils have also been demonstrated to contribute to tumour angiogenesis employing similar mechanisms.^30^

Here, we report that macrophage GPR35 is important in vessel growth in healthy and malignant tissue. GPR35 critically controls the size of intestinal tumours in murine spontaneous (APC*^min^*) and CAC (AOM/DSS) models. We demonstrate that deletion of *Gpr35* in macrophages recapitulates the profoundly reduced tumour size observed in *Gpr35* ^–/–^ germline mutant mice, identifying macrophage GPR35 as a key driver of tumour growth. Aortic tissue from mice lacking GPR35 is strikingly less capable of developing vascular sprouts. Vessel sprouting is mainly dependent on GPR35’s interaction with the Na/K-ATPase-dependent activation of Src kinase, and does not require ligand-induced GPR35 activation.

## Materials and methods

For detailed resources, please see online supplemental materials and methods and online supplemental table 1.

10.1136/gutjnl-2020-323363.supp1 Supplementary data

## Results

### Macrophage GPR35 promotes tumour growth

**Figure 1:**
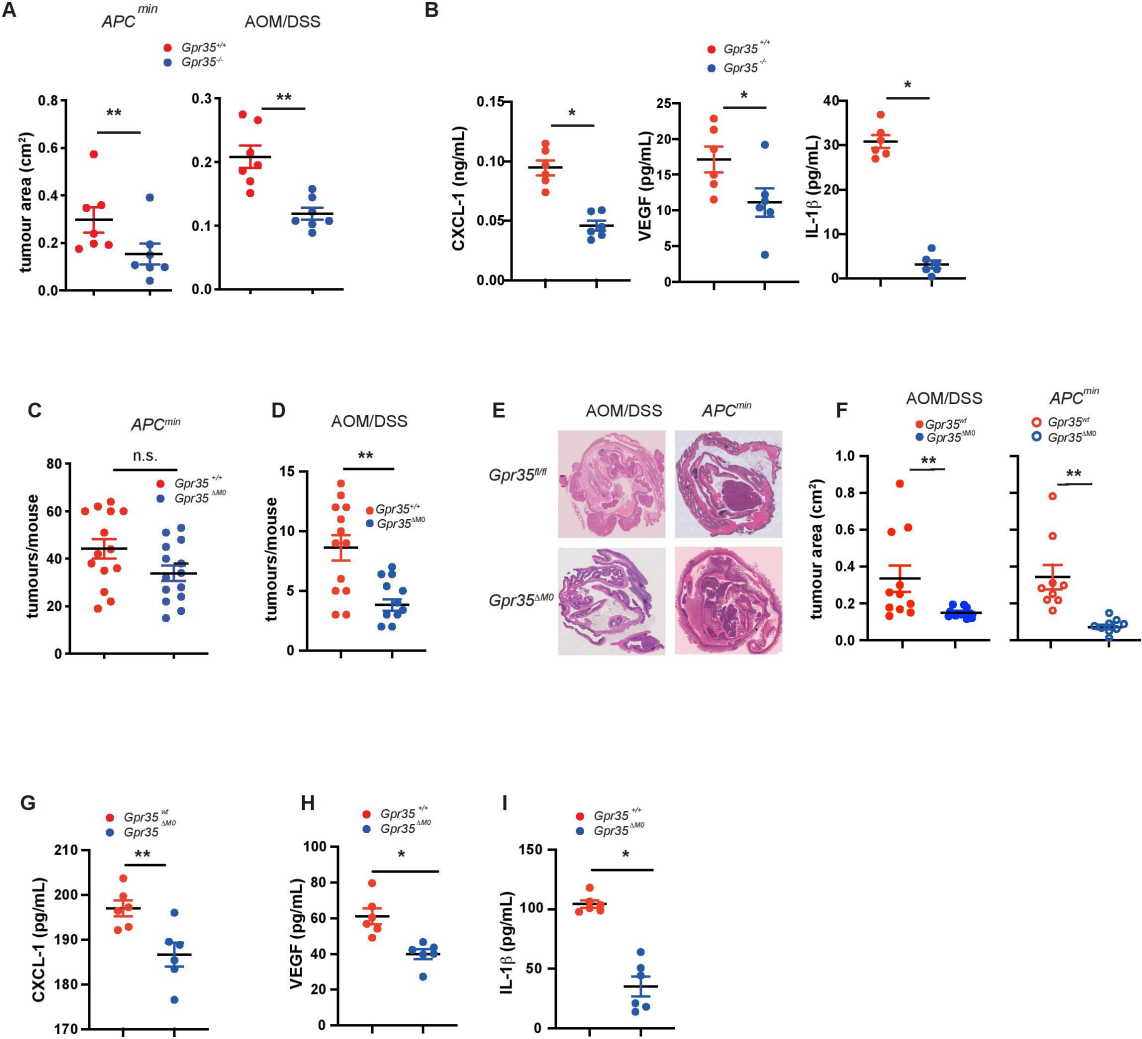
GPR35 increases tumour size and release of proangiogenic cytokines. (A) The area of intestinal adenomas from Apc*^min^* mice either wild-type (*Gpr35^+/+^*) and globally deficient for GPR35 (*Gpr35^-/-^*) (left panel) or from *Gpr35^+/+^* and *Gpr35^-/-^* mice exposed to AOM and three cycles of DSS (right panel) were compared. N=7 tumours per genotype. (B) CXCL-1, VEGF and IL-1β levels measured in AOM/DSS tumour tissue of *Gpr35^+/+^* and *Gpr35^-/-^* mice. N=6 tumours per genotype. (C) Mice conditionally deficient for macrophage GPR35 (*Gpr35* ^ΔMΦ^) were crossed with *APC^min^* mice and tumours were counted when the mice reached an age of 16 weeks. N=14 mice per genotype. (D) *Gpr35* ^ΔMΦ^ mice were exposed to AOM and DSS. Colon adenomas were counted in *Gpr35* ^ΔMΦ^ mice and in their control *Gpr35^fl/fl^*. N=11 to 13 mice per genotype. (E) Representative images showing H&E stain of tumours from *Gpr35^fl/fl^* and *Gpr35* ^ΔMΦ^ mice. (F) The area of intestinal adenomas from either *Gpr35^fl/fl^*; APC*^min^* and *Gpr35* ^ΔMΦ^; *Apc* ^min^ mice (left panel) or from *Gpr35^fl/fl^* and *Gpr35* ^ΔMΦ^ mice exposed to AOM/DSS (right panel) were compared. N=9 tumours per genotype. (G–I) CXCL-1 (G), VEGF (H) and IL-1β (I) levels measured in AOM/DSS tumour tissue of *Gpr35^fl/fl^* and *Gpr35* ^ΔMΦ^ mice. N=6 tumours per genotype. All data represented as mean±SEM. Statistical significance was calculated using Mann-Whitney U after Kruskal-Wallis testing. * p<0.05, ** p<0.01. AOM, azoxymethane; APC, Adenomatosis Polyposis Coli; DSS, dextran sodium sulphate; IL-1β, interleukin 1β; n.s., not significant.

We had previously reported a reduced incidence of intestinal tumours in *Gpr35* ^–/–^ mice in spontaneous CRC (*Apc* ^min^) and CAC (AOM/DSS) models.^10^ Selective deletion of *Gpr35* in the intestinal epithelium (ie, in *Gpr35* ^fl/fl^;*Vil*-Cre or ‘*Gpr35* ^ΔIEC^’ mice) was sufficient to reduce tumour incidence in both models.^10^ In both CRC and CAC, individual tumours in *Gpr35* ^–/–^ mice were strikingly smaller compared with *Gpr35* ^+/+^ tumours (figure 1A). Levels of CXCL-1, VEGF and IL-1β, proangiogenic mediators mainly produced by TAM,^28 31 32^ were substantially lower in *Gpr35* ^–/–^ compared with *Gpr35* ^+/+^ tumours (figure 1B). This prompted us to ask whether GPR35 on LysM^+^ cells, which encompasses macrophages and neutrophils,^33^ might mediate a TME pathway that promotes tumour growth.^17 18^ We, therefore, generated *Apc* ^min^ allele-carrying mice with LysM-specific *Gpr35* deletion (*Gpr35* ^fl/fl^;LysM-*Cre;Apc* ^min/+^, ‘*Gpr35* ^ΔMΦ^;*Apc* ^min^’) and compared them to their *Gpr35*-sufficient controls (*Gpr35* ^fl/fl^;*Apc* ^min/+^, ‘*Gpr35* ^fl/fl^;*Apc* ^min^’) (figure 1E). *Gpr35* ^ΔMΦ^;*Apc* ^min^ mice developed tumours more than three times smaller than *Gpr35* ^fl/fl^;*Apc* ^min^ mice (figure 1F), although the total number of tumours was not significantly different (figure 1C). Subjecting *Gpr35* ^ΔMΦ^ (ie, without an *Apc* ^min^ allele) to AOM/DSS colitis revealed a similarly stark reduction in tumour size compared with their *Gpr35* ^fl/fl^ controls (figure 1F). In this model of CAC, even the total tumour count in *Gpr35* ^ΔMΦ^ mice was half of that in *Gpr35* ^fl/fl^ mice (figure 1D), and associated with a higher survival rate (online supplemental figure 1D). The reduced incidence and size of tumours in *Gpr35* ^ΔMΦ^ mice was not linked to protection from DSS-induced colitis, as weight loss during short-term (ie, 7 days) DSS, nor during the three cycles of AOM/DSS was indifferent between *Gpr35* ^+/+^ and *Gpr35* ^–/–^, and *Gpr35* ^fl/fl^ and *Gpr35* ^ΔMΦ^ mice (online supplemental figure 1A–C). Levels of VEGF, CXCL-1 and IL-1β were also markedly lower in inflammatory AOM/DSS tumours of *Gpr35* ^ΔMΦ^ compared with *Gpr35* ^fl/fl^ mice (figure 1G–I). Inflammatory infiltrate was reduced in *Gpr35* ^ΔMΦ^ tumours compared with *Gpr35* ^fl/fl^ tumours (online supplemental figure 1E). Macrophages and neutrophils express *Gpr35*, which is efficiently deleted in both cell types in *Gpr35* ^ΔMΦ^ mice (online supplemental figure 2B). Of note, *GPR35* expression extends to human peripheral blood-derived neutrophils and monocyte-derived macrophages (online supplemental file 2C). Neutrophil activation, measured by tissue MPO levels, were similar in *Gpr35* ^ΔMΦ^ and *Gpr35* ^fl/fl^ mice after 3 cycles of AOM/DSS, suggesting neutrophil GPR35 did not affect neutrophil activation per se in this context (online supplemental figure 1F). Altogether, this demonstrated that GPR35 in LysM^+^ myeloid cells potently promotes tumour growth in both CRC and CAC, and hinted toward an important role of tumour angiogenesis in this microenvironmental pathway.

10.1136/gutjnl-2020-323363.supp2 Supplementary data

### GPR35 deletion in LysM^+^ myeloid cells reduces angiogenesis

**Figure 2:**
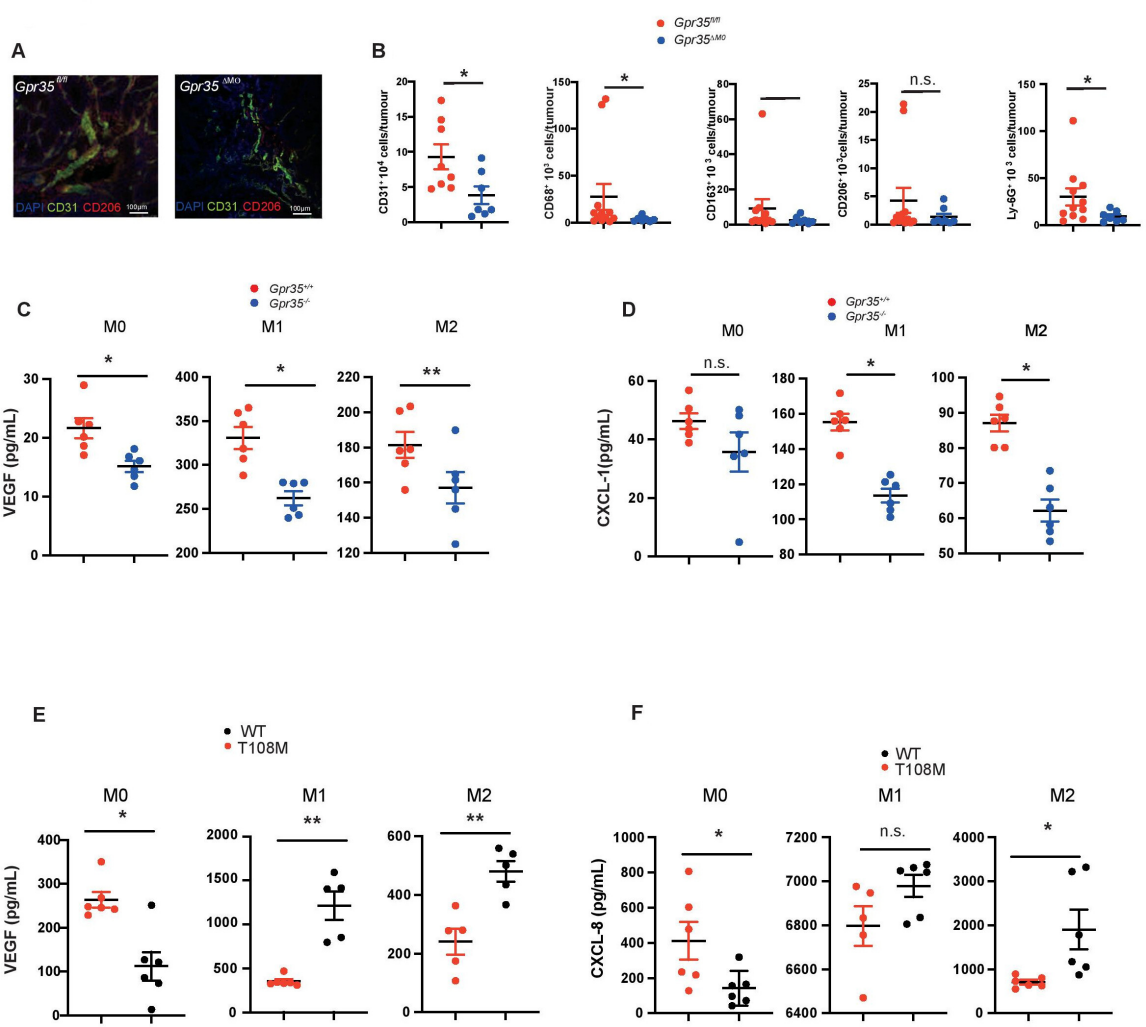
GPR35 deletion in myeloid cells reduces angiogenic potential. (A) Adenomas from *Gpr35^fl/fl^ and Gpr35* ^ΔMΦ^ mice exposed to AOM/DSS stained for CD31^+^ endothelial cells and CD206^+^ M2 macrophages. N=5 each genotype, representative confocal microscopy. Scale bars 100 µm. (B) FACS analyses of CD31^+^, CD68^+^, CD163^+^, CD206^+^ and Ly-G6^+^ cells in *Gpr35^fl/fl^* and *Gpr35* ^ΔMΦ^ tumour tissue. N=8–12 *Gpr35^fl/fl^* tumours from 8 to 12 mice and N=7 *Gpr35* ^ΔMΦ^ tumours from mice. (C) VEGF levels in M0, M1 and M2 BMDM from *Gpr35^+/+^ and Gpr35^-/-^* mice. N=6 per genotype. (D) CXCL-1 levels in M0, M1 and M2 BMDM from *Gpr35^+/+^ and Gpr35^-/-^* mice. N=6 per genotype. (E) VEGF levels in supernatants of M0, M1 and M2 human iPS cell-derived macrophages. N=6 each genotype. (F) CXCL-8 levels in supernatants of M0, M1 and M2 human iPS cell-derived macrophages. N=6 each genotype. * p<0.05, ** p<0.01. AOM/DSS, azoxymethane followed by dextran sodium sulphate; iPS, inducible-pluripotent-stem; n.s., not significant; WT, wild type.

**Figure 3:**
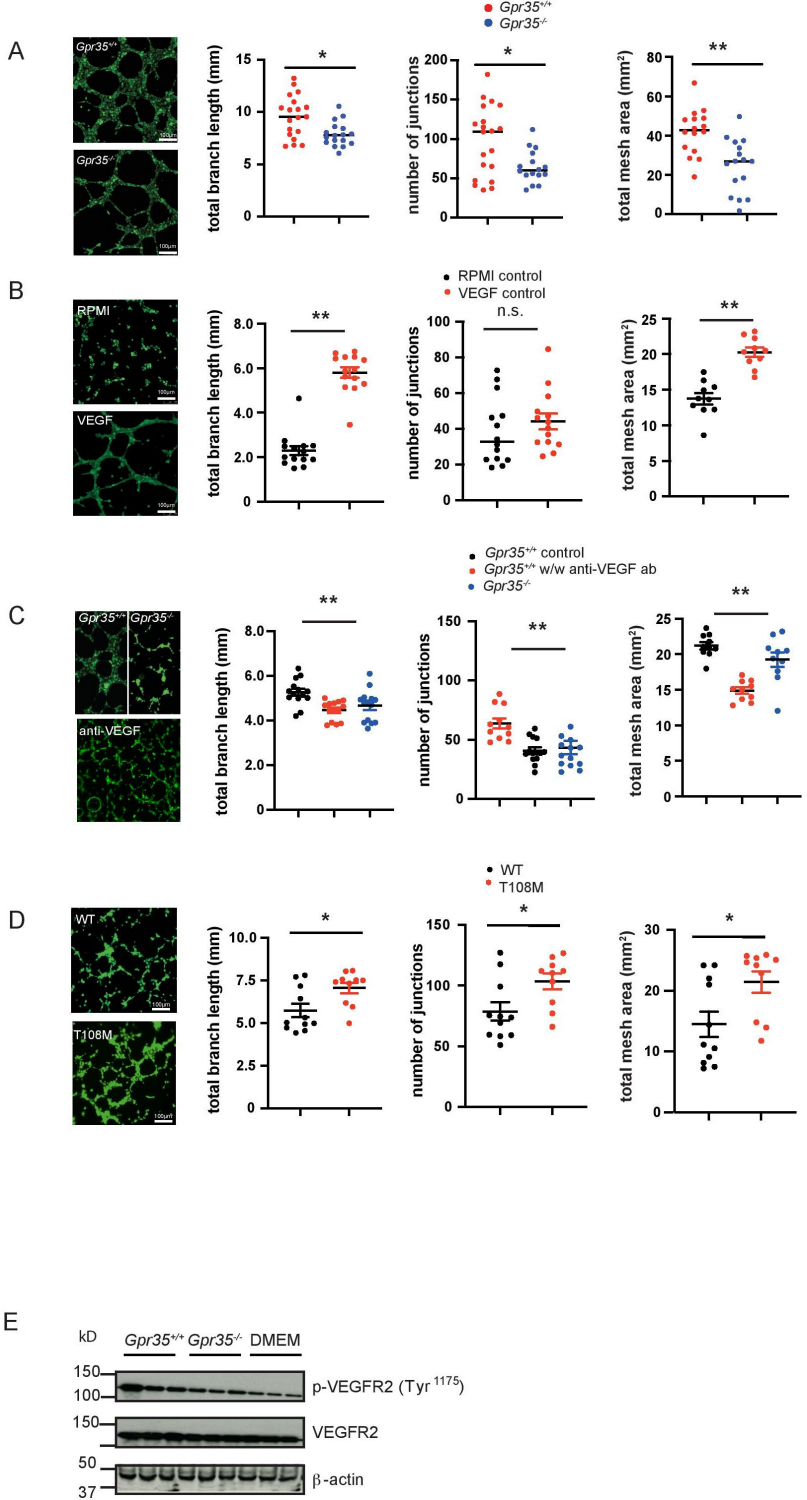
GPR35 deletion in macrophages and neutrophils reduces tube formation. (A) Tube formation assay with murine SVEC endothelial cells incubated with *Gpr35^+/+^* and *Gpr35^-/-^* M2 macrophage supernatants. Branch length, number of junctions and total mesh area were determined using ImageJ’s angiogenesis tool. N=18. (B) 2 H-11 control tube formation assays with control RPMI/10%FBS and RPMI/10%FBS containing 30 ng/mL VEGF. No macrophage supernatants present. Branch length, number of junctions and total mesh area were determined using the Image J’s angiogenesis tool. N=14 C. 2 H-11 tube formation assays with supernatants of wild-type (WT) and knock-out macrophages in the presence of blocking anti-VEGF antibody. Branch length, number of junctions and total mesh area were determined using Image J’s angiogenesis tool. N=14. (D) HMVEC tube formation assays with human microvascular endothelial cells. Endothelial cells were incubated with supernatants of iPS cell-derived macrophage supernatants (WT and T108M risk variant). Branch length, number of junctions and total mesh area were determined using Image J’s angiogenesis tool. N=10. (E) VEGF receptor 2 phosphorylation of 2 H-11 cells treated with WT or *Gpr35^–/–^* M2 supernatants. All data represented as mean±SEM. Statistical significance was calculated using Mann-Whitney U after Kruskal-Wallis testing. HMVEC, human microvascular endothelial cell; iPS, inducible-pluripotent-stem; n.s., not significant.

The architecture of blood vessels in AOM/DSS-induced tumours of *Gpr35* ^fl/fl^ mice appeared to be different from that in tumours of *Gpr35* ^ΔMΦ^ mice. We observed fewer endothelial cells, identified by CD31, which stretched along the adenomatous crypts in *Gpr35* ^ΔMΦ^ mice, whereas in adenomas from *Gpr35* ^fl/fl^ mice, the CD31^+^ neo-vasculature resembled a reticular structure (figure 2A). When these tumours were analysed for total numbers of CD31^+^ cells by FACS, fewer CD31^+^ cells were detected in *Gpr35* ^ΔMΦ^ compared with *Gpr35* ^fl/fl^ tumours (figure 2B). Neither primary CD31^+^ murine endothelial cells, nor human umbilical vein endothelial cells, human microvascular endothelial cells (HMVECs), or the murine endothelial cell lines SVEC40 and H2-11 expressed *Gpr35* mRNA (online supplemental figure 2A). We also noticed that adenomas in *Gpr35* ^ΔMΦ^ mice exhibited only sparse staining for CD206, the mannose receptor and a marker of ‘M2’ macrophages, compared with dense staining in *Gpr35* ^fl/fl^ adenomas (figure 2A). FACS analysis of *Gpr35* ^ΔMΦ^ tumours (online supplemental figure 2D) showed significantly less CD68^+^ cells compared with *Gpr35* ^fl/fl^ tumours (figure 2B). The number of CD163^+^ and CD206^+^ cells also trended lower in *Gpr35* ^ΔMΦ^ compared with *Gpr35* ^fl/fl^ tumours (figure 2B). Ly6-G^+^ cell counts were lower in *Gpr35* ^ΔMΦ^ compared with *Gpr35* ^fl/fl^ tumours, consistent with lower immune cell infiltration (online supplemental figure 1E.) Bone-marrow derived *Gpr35* ^+/+^ macrophages stimulated to differentiate into paradigmatic ‘M0’, ‘M1’ and ‘M2’ phenotypes secreted substantially more of the angiogenic mediators VEGF and CXCL-1 compared with their *Gpr35* ^–/–^ counterparts (figure 2C, D). Human M0, M1 and M2 macrophages, differentiated from induced pluripotent stem cells (iPSC), secreted substantially more VEGF and CXCL-8 when they were differentiated from iPSCs genetically engineered to express the hypermorphic GPR35 T108M risk variant,^10^ compared with those differentiated from their syngeneic non-risk parent strain (figure 2E, F). This demonstrated that GPR35-dependent angiogenic factor release is conserved across species, and the human risk variant profoundly enhances their secretion. Macrophages involved in repair and (tumour)angiogenesis are thought to resemble mainly an M2 phenotype. We, therefore, pursued endothelial tube formation assays with SVECs or H2-11 murine endothelial cells stimulated with supernatants from *Gpr35* ^+/+^ and *Gpr35* ^–/–^ M2 macrophages. Conditioned media from *Gpr35* ^+/+^ M2 macrophages induced SVECs to form significantly longer branches, more junctions within the tubular network, and a total area of formed tubes significantly larger compared with conditioned media from *Gpr35* ^–/–^ macrophages (figure 3A). This suggested that GPR35 controls the release of angiogenic factors in macrophages that promote endothelial tube formation, a prerequisite of neovascularisation. Tube formation only occurs in the presence of growth factors such as VEGF. Control experiments show that supernatants of BMDM contain sufficient amounts of VEGF and potential other soluble factors CXCL-1/CXCL-8. Control RPMI media was not able to induce tube formation in 2 H-11 cells, which recurred on addition of recombinant VEGF (figure 3B). Blocking VEGF with an anti-VEGF antibody reduced branch length, number of junctions and the total mesh area, indicating the importance of growth factors released by macrophages (figure 3C). Increased branching and mesh formation of 2 H-11 endothelial cells (online supplemental figure 3) when exposed to *Gpr35* ^+/+^ compared to *Gpr35* ^–/–^ macrophage supernatants was associated with increased phosphorylation at Tyr^1175^ of the VEGF receptor (figure 3E and online supplemental figure 5A). HMVEC exposed to supernatants from human T108M M2 macrophages differentiated from iPSCs also exhibited increased branching and mesh formation compared with those from their non-risk counterparts (figure 3D). These data were consistent with GPR35-dependent secretion of factors, including VEGF, from macrophages that act on endothelial cells to promote vessel formation.

### GPR35 promotes ex vivo endothelial sprouting

**Figure 4:**
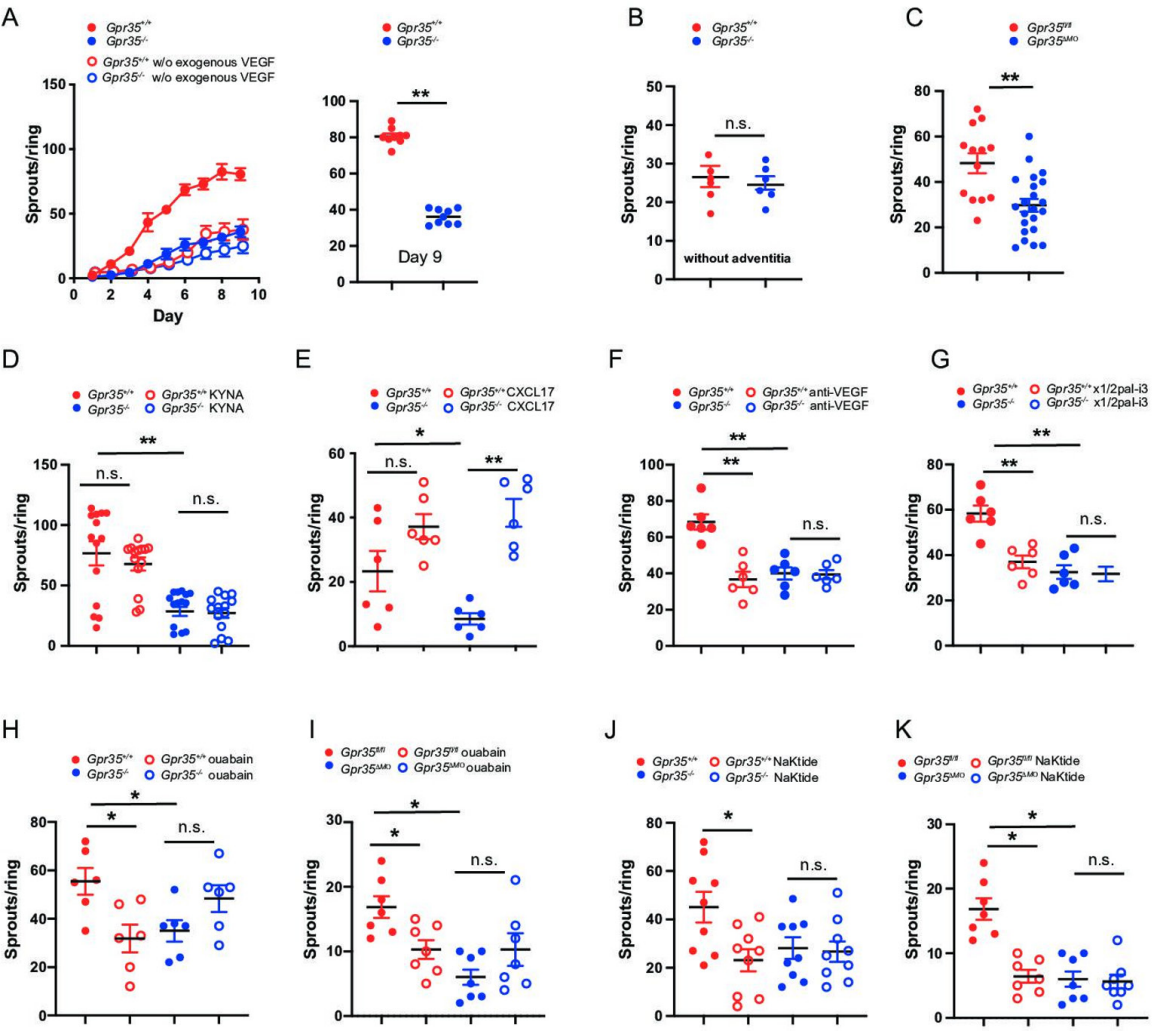
Macrophage GPR35 controls vascular sprouting by interacting with the Na/K-ATPase. (A) Aortic rings from *Gpr35* ^+/+^ or *Gpr35* ^–/–^ mice embedded in collagen matrix. Number of sprouts counted daily until day 9 of the experiment. Aortic rings were either embedded in media containing 30 ng/mL of VEGF (full circles) or left in OptiMEM media without growth factors (open circles) N=9 mice per genotype. (B) Vascular adventitia was removed from aortic rings from *Gpr35* ^+/+^ or *Gpr35* ^–/–^. Number of sprouts on day 9. N=6 mice per genotype. (C) Aortic rings from wildtype animals (*Gpr35^fl/fl^*) or mice lacking GPR35 conditionally on their LysM^+^ cells*Gpr35* ^ΔMΦ^. N=13 for *Gpr35^fl/fl^* and 23 for Gpr35^ΔMΦ^ mice. (D) KYNA (100 µM) was added to the aortic rings of *Gpr35* ^+/+^ or *Gpr35* ^–/–^ mice and vascular sprouts counted on day 9. N=5 mice for each genotype. (E) CXCL-17 (20 ng/mL) was added to the aortic rings of *Gpr35* ^+/+^ or *Gpr35* ^–/–^ mice and vascular sprouts counted on day 9. N=6 mice for each genotype. (F) Anti-VEGF antibody (100 ng/mL) was added to the aortic rings of *Gpr35* ^+/+^ or *Gpr35* ^–/–^ mice and vascular sprouts counted on day 9. n=6 for each genotype. (G) Blocking CXCR2 pepducin x1/2pal-i3 (3 µM) was added to the aortic rings of *Gpr35* ^+/+^ or *Gpr35* ^–/–^ mice and vascular sprouts counted on day 9. n=6 for each genotype. (H, I.) ouabain (100 µM) was added to the aortic rings of *Gpr35* ^+/+^ or *Gpr35* ^–/–^ mice (H) and to the aortic rings of *Gpr35^fl/fl^* and *Gpr35* ^ΔMΦ^ mice (I) and vascular sprouts counted on day 9. N=6 for each genotype. (J, K) pNaKtide (1 µM) was added to the aortic rings of *Gpr35* ^+/+^ or *Gpr35* ^–/–^ mice (J) and to the aortic rings of *Gpr35^fl/fl^* and *Gpr35* ^ΔMΦ^ mice (K) and vascular sprouts counted on day 9. N=9 for each genotype. All data represented as mean±SEM. Statistical significance was calculated using Mann-Whitney U after Kruskal-Wallis testing.* p<0.05, ** p<0.01. n.s., not significant.

On in vitro culture, fibroblasts and macrophages are the first cells to migrate out of the aortic ring, setting the stage for the formation of neovessels. Endothelial cells typically start migrating and proliferating on day 2–3, and pericytes eventually surround the elongating sprouts.^34 35^ Here, we tested whether GPR35 is involved in vascular sprouting in vascular tissue and hence subjected aortic rings from wild-type and knock-out mice to an aortic sprout assay. *Gpr35* ^–/–^ aortic rings developed markedly fewer vessel sprouts compared with *Gpr35* ^+/+^ rings (figure 4A). Addition of exogenous VEGF (30 ng/mL) was required (added on day 1), as only very little sprouting was triggered in wild-type and knock-out aortic rings in its absence (figure 4A). Removal of large parts of the aortic adventitia, which contains macrophages, abrogated the difference between *Gpr35* ^+/+^ and *Gpr35* ^–/–^ aortic rings and let overall far fewer sprouts develop compared with when the aortic wall was left intact (figure 4B). Vessel sprouting from *Gpr35* ^ΔMΦ^ compared with *Gpr35* ^fl/fl^ aortic rings was equally profoundly suppressed (figure 4C) as in *Gpr35^–/–^* compared with *Gpr35* ^+/+^ aortic rings. This demonstrated that GPR35 on LysM^+^ myeloid cells orchestrated the outgrowth of neovessels.

KYNA, a product of tryptophan catabolism, and the chemokine CXCL-17 have been suggested as ligands of GPR35.^36 37^ Recombinant CXCL-17, added to aortic ring cultures, increased vessel sprouting in both *Gpr35* ^+/+^ and *Gpr35* ^–/–^ aortic rings, indicating that CXCL-17 signals in a GPR35-independent manner (figure 4E). KYNA did not alter vessel sprouting, suggesting it is not involved in GPR35-mediated angiogenesis (figure 4D). Addition of a neutralising anti-VEGF monoclonal antibody on day 2 of the experiment, that is, after initial exogenous triggering, reduced vessel sprouting in *Gpr35* ^+/+^ aortic rings to levels observed in *Gpr35* ^–/–^ aortic rings, in which sprouting was not affected (figure 4F). Moreover, blocking CXCL-1 signalling by blocking its receptor CXCR2 with a selective pepducin^38^ also reduced vessel sprouting from *Gpr35* ^+/+^, but not from *Gpr35* ^–/–^ aortic rings (figure 3G). This demonstrated that GPR35 promotes vessel sprouting via VEGF and CXCR2, which tallied with higher VEGF and CXCL-1 secretion in tissue and macrophages from *Gpr35* ^+/+^ compared to *Gpr35* ^–/–^ mice.

### GPR35 promotes angiogenesis via the sodium-potassium-pump in macrophages

Reduced Na/K-ATPase ion pumping activity in the absence GPR35 elevates intracellular Ca^2+^ and lowers K^+^ levels.^10^ The endogenous cardiotonic steroid ouabain achieves a similar effect via binding to Na/K-ATPase and retaining it in an inactive (E2P) state.^39^ Ouabain indeed reduced vessel sprouting in *Gpr35* ^+/+^ aortas to levels observed in *Gpr35* ^–/–^ aortas, whereas sprouting was not significantly changed but slightly increased in *Gpr35* ^–/–^ aortas (figure 4H). This was precisely phenocopied in aortas from *Gpr35* ^fl/fl^ when compared with *Gpr35* ^ΔMΦ^ mice (figure 4I), demonstrating vessel sprouting was coordinated by GPR35 on myeloid cells in a Na/K-ATPase-dependent manner. Among its non-pump functions, Na/K-ATPase acts as a scaffold for Src kinase and is thereby important in signal transduction.^11 40 41^ The α1 type Na/K-ATPase forms a complex with the Src SH2 and kinase domain and keeps the kinase domain in an inactive state.^11^ A 20-amino acid sequence (pNaKtide) specifically inhibits Na/K-ATPase-associated Src activation.^42 43^ Addition of pNaKtide peptide decreased sprouting in *Gpr35* ^+/+^ and *Gpr35* ^fl/fl^ aortas to levels observed in their respective *Gpr35* ^–/–^ and *Gpr35* ^ΔMΦ^ counterparts, in which pNaKtide had no further effect (figure 4J, K). When exposing *Gpr35* ^+/+^ macrophages to ouabain, no increase in Src phosphorylation was observed. This was unexpected given previously published work in cell lines that do not endogenously express GPR35.^43^ Src phosphorylation was indeed increased by ouabain in *Gpr35* ^–/–^ macrophages (online supplemental figure 4), pointing towards an important role of GPR35 determining the Src signalling response on pharmacological inhibition of Na/K-ATPase. Altogether this demonstrated that GPR35 expressed on myeloid cells within the aortic wall promotes vessel sprouting in a manner dependent on Na/K-ATPase-triggered Src activation.

### GPR35-deficient tumours produce less MMPs

**Figure 5:**
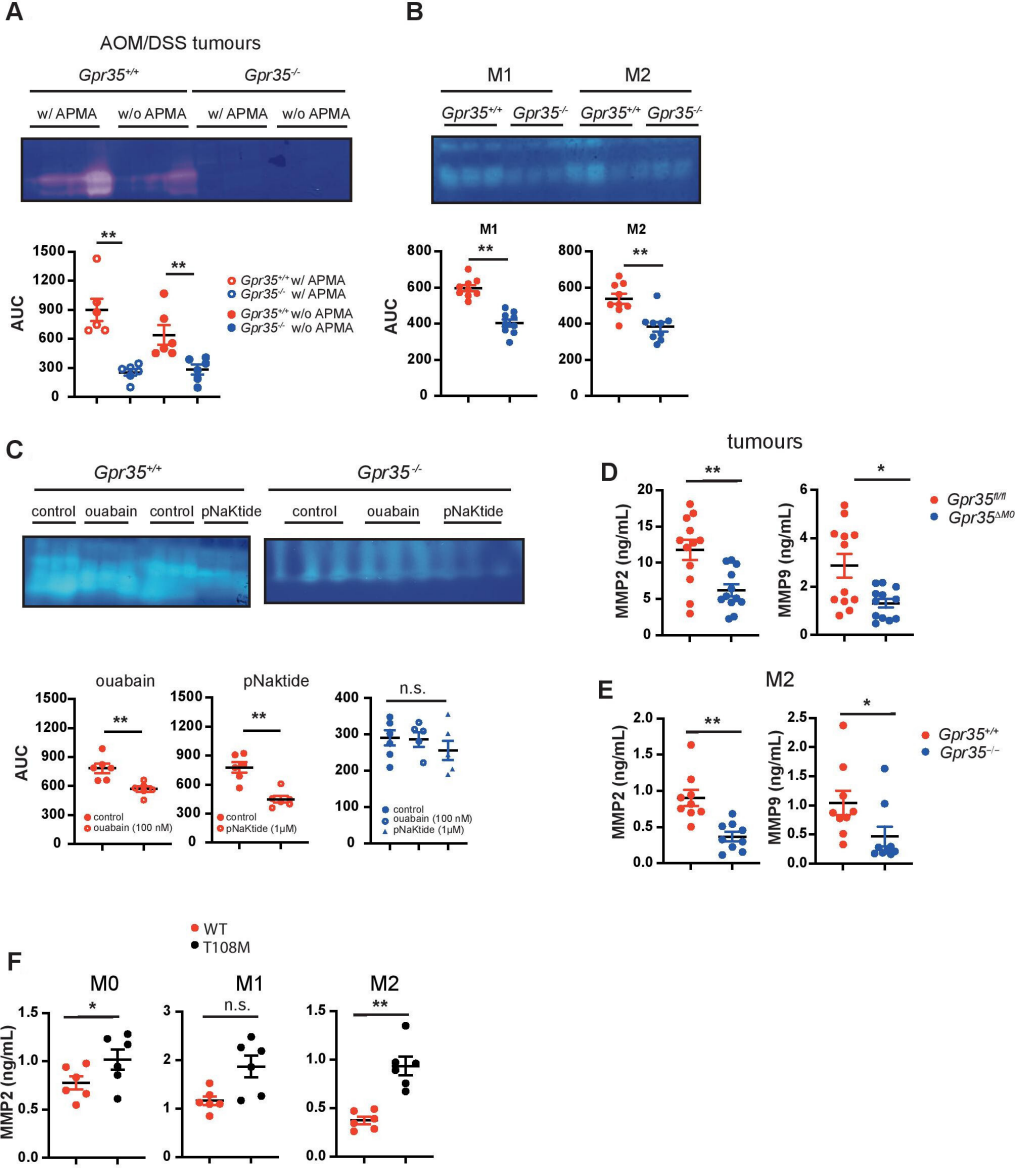
Lack of GPR35 results in reduced MMP levels. (A) MMP levels in *Gpr35* ^+/+^ and *Gpr35* ^–/–^ adenomas. Tissue extract MMPs were activated with APMA. Area under the curve was analysed using image J. N=6 tumours per genotype. (B) MMP levels in M1 and M2 BMDM supernatants from *Gpr35* ^+/+^ and *Gpr35* ^–/–^ mice. Supernatants were APMA-activated. area under the curve was analysed using image J N=9 mice per genotype. (C) MMP levels in M2 macrophages pretreated with ouabain (100 µM) or pNaKtide (1 µM). Area under the curve was analysed using image J N=6 mice per genotype. (D) Total (inactive zymogen and active enzyme) MMP2 and MMP9 levels in tumour tissue from *Gpr35^fl/fl^* and *Gpr35* ^ΔMΦ^ mice. (E) MMP2 and MMP9 levels from M2 murine BMDM supernatants. (F) Total (inactive zymogen and active enzyme) MMP2 levels from supernatants of human iPS cell-derived macrophages. Macrophages were polarised to M0, M1 and M2 macrophages. All data represented as mean±SEM. Statistical significance was calculated using Mann-Whitney U after Kruskal-Wallis testing. * p<0.05, ** p<0.01/ APMA, aminophenylmercuric acetate; MMP, matrix-metallo-proteinase; n.s., not significant; WT, wild type.

A link between MMP-9-positive TAMs and endothelial cells responding to angiogenic induction has been established.^44^ To assess MMP activity, we performed gelatine zymography of extracts from tumours arising in the AOM/DSS model. *Gpr35* ^–/–^ tumours exhibited much lower gelatinolytic activity compared with *Gpr35* ^+/+^ tumours (figure 5A). Addition of 4-aminophenylmercuric acetate (APMA), which activates pro-MMPs to active zymogens, to tumour extracts did not further increase their gelatinolytic activity (figure 5A). This indicated that essentially all available MMP activity in tumours is already in the active form. Macrophages can release MMPs and tissue inhibitors of MMPs. While native supernatants from *Gpr35* ^–/–^ or *Gpr35* ^+/+^ M1 and M2 BMDMs did not exhibit MMP activity (data not shown), addition of APMA to supernatants revealed substantial activity in *Gpr35* ^+/+^ and much lower activity in *Gpr35* ^–/–^ macrophage supernatants (figure 5B). Ouabain and pNaKtide treatment of *Gpr35* ^+/+^ M2 macrophages reduced their supernatants’ MMP activity (figure 5C), whereas it did not affect MMP activity in *Gpr35* ^–/–^ supernatants (figure 5C). Differences in zymography were detected in gelatine- but not casein gels. We, therefore, measured the gelatinases MMP2 and MMP9 in tumour tissue and supernatants from BMDM. *Gpr35* ^ΔMΦ^ tumours contained substantially less MMP2 and MMP9 (figure 5D) compared with AOM/DSS-induced tumours from *Gpr35^fl/fl^* mice. M2 macrophages from *Gpr35* ^–/–^ mice correspondingly contained less MMP2 and MMP9 compared with their *Gpr35* ^+/+^ counterparts (figure 5E). Moreover, supernatants from human iPSC-derived M0 and M2 macrophages carrying the T108M risk variant contained higher amounts of MMP2 compared with those from non-risk variant cells, with MMP2 levels in T108M variant M1 macrophages levels also trending higher (figure 5F). Altogether, this implied that GPR35 controlled via Na/K-ATPase-dependent Src activation the level of MMP activity in tumours, with the T108M variant yielding highest levels.

### GPR35 increases endothelial transmigration of monocytes

**Figure 6:**
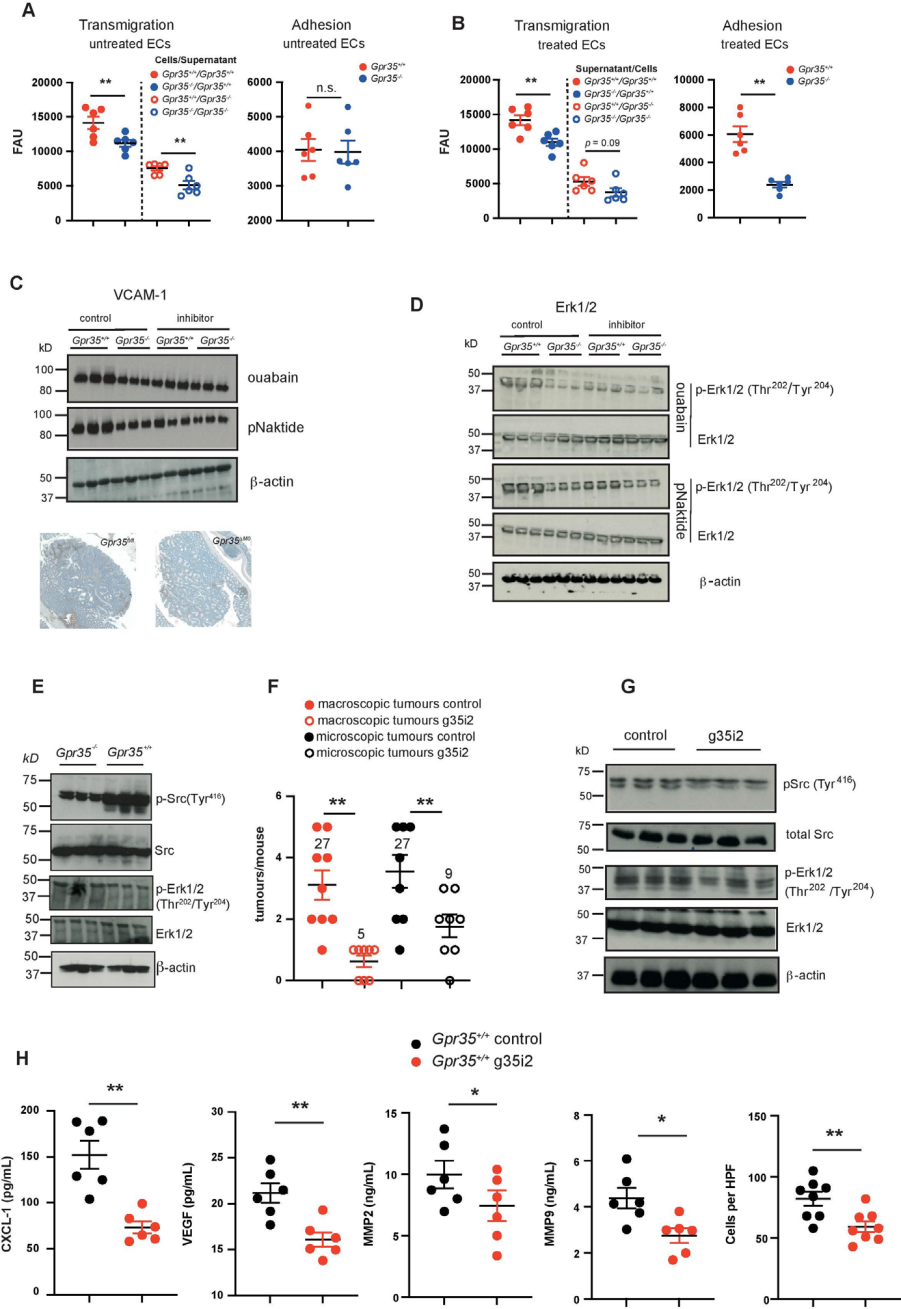
Lack of GPR35 decreases endothelial transmigration. (A) Right panel: *Gpr35* ^+/+^ and *Gpr35* ^–/–^ monocytes transendothelial migration towards conditioned media from *Gpr35* ^+/+^ and *Gpr35* ^–/–^ M2 macrophages in the lower compartments of migration chambers. Untreated endothelial cell monolayer. Left panel: adhesion of *Gpr35* ^+/+^ and *Gpr35* ^–/–^ monocytes to 2 H-11 murine endothelial cells. Untreated endothelial cell monolayer. N=6 mice per genotype. (B) Left panel: transendothelial migration of monocytes through monolayers of 2 H-11 murine endothelial cells exposed to conditioned media from *Gpr35* ^+/+^ and *Gpr35* ^–/–^ M2 macrophages. right panel: adhesion of *Gpr35* ^+/+^ and *Gpr35* ^–/–^ monocytes to 2 H-11 murine endothelial cells treated with conditioned media from *Gpr35* ^+/+^ and *Gpr35* ^–/–^ M2 macrophages. N=6 mice per genotype. (C) Upper panel: VCAM-1 expression in 2 H-11 cells treated with conditioned media from *Gpr35* ^+/+^ and *Gpr35* ^–/–^ M2 macrophages pretreated with control RPMI, ouabain (100 µM) or pNaKtide (1 µM). Lower panel: VCAM-1 expression in *Gpr35^fl/fl^* and *Gpr35* ^ΔMΦ^ AOM/DSS tumours. (D) ERK1/2 phosphorylation of 2 H-11 cells treated with conditioned media from *Gpr35* ^+/+^ and *Gpr35* ^–/–^ M2 macrophages pretreated with control RPMI, ouabain (100 µM) or pNaKtide (1 µM). (E) Src and ERK1/2 phosphorylation in *Gpr35* ^+/+^ and *Gpr35* ^–/–^ AOM/DSS tumours. N=3 tumours per genotype. (F) Numbers of macroscopic and microscopic tumours of either g35i2 or vehicle treated mice with AOM/DSS induced tumours. N=8–9 mice/group. (G) AOM/DSS tumour tissue from g35i2 or vehicle treated mice probed for Src and ERK1/2 phosphorylation. N=3 tumours per genotype. (H) CXCL-1, VEGF, MMP2, MMP9 and inflammatory infiltrate in tumour tissue from g35i2 or vehicle-treated wild-type mice. N=6 per genotype for CXCL-1, VEGF, MMP2 and MMP9 ELISA assays, and n=8 for histological assessment of inflammatory cell infiltrate. All data represented as mean±SEM. Statistical significance was calculated using Mann-Whitney U after Kruskal-Wallis testing. AOM/DSS, azoxymethane followed by dextran sodium sulphate; n.s., not significant.

Circulating CCR2^+^ inflammatory monocytes enter the TME, proliferate and differentiate into TAMs,^45^ a process that requires endothelial adhesion and transmigration.^46^ We therefore investigated transmigration by studying myeloid cells harvested from 5-day bone marrow cultures, at which point cells exhibit a monocyte phenotype.^47^ Mimicking monocyte transmigration, we plated endothelial cells onto Transwell membranes and used supernatants from *Gpr35* ^+/+^ or *Gpr35* ^–/–^ M2 macrophages as chemoattractant. *Gpr35* ^+/+^ or *Gpr35* ^–/–^ monocytes added to the upper compartment of the chamber migrated through the endothelial cell layer towards the macrophage supernatants. Compared with *Gpr35* ^+/+^ monocytes, transmigration of *Gpr35* ^–/–^ monocytes was reduced (figure 6A, left panel). Fewer *Gpr35* ^–/–^ than *Gpr35* ^+/+^ monocytes adhered to monolayers of H2-11 murine endothelial cells (figure 6A, right panel). Hence GPR35 deficiency in monocytes impaired their ability to adhere to, and transmigrate through, endothelial cells. In converse experiments, we exposed endothelial cells to supernatants of either *Gpr35* ^+/+^ or *Gpr35* ^–/–^ M2 macrophages, and monocytes of either genotype migrated towards wild-type macrophage conditioned media. Monocytes adhered less to endothelial cells that were exposed to *Gpr35* ^–/–^ M2 macrophage supernatant than to those exposed to *Gpr35* ^+/+^ supernatant (figure 6B right panel). Transmigration of monocytes through an endothelial monolayer incubated with *Gpr35* ^–/–^ M2 macrophage conditioned media was correspondingly reduced when compared with migration through endothelial cells exposed *Gpr35* ^+/+^ supernatants. Fewer *Gpr35* ^–/–^ than *Gpr35* ^+/+^ monocytes migrated through monolayers treated with either *Gpr35* ^–/–^ or *Gpr35* ^+/+^ macrophage supernatants (figure 6B, left panel). This demonstrated that GPR35 on macrophages controls the release of factors that promoted adhesion and transendothelial migration of monocytes.

VCAM-1 is a major endothelial cell adhesion molecule that facilitates monocyte transmigration from the peripheral blood into the surrounding tissue.^48^ VCAM-1 expression was higher in H2-11 endothelial cells exposed to *Gpr35* ^+/+^ compared with *Gpr35* ^–/–^ M2 macrophage supernatants (figure 6C). This increase was abrogated when M2 macrophages were treated overnight with ouabain or pNaKtide and then washed, prior to collecting supernatants over the ensuing 24 hours (figure 6C and online supplemental figure 5B). In contrast, ouabain or pNaKtide treatment of *Gpr35* ^–/–^ M2 macrophages did not affect endothelial VCAM-1 expression. This demonstrated that increased VCAM-1 induction on endothelial cells was dependent on GPR35, Na/K-ATPase and Src activity in macrophages. Consistent with such GRP35-dependent regulation, VCAM-1 expression in tissue was higher in *Gpr35^fl/fl^* compared with *Gpr35* ^ΔMΦ^ mice (figure 6C). Activation of extracellular signal-regulated kinases (ERK) 1 and 2 by VCAM-1 is critical for endothelial activation, transmigration and angiogenesis.^48^ Conditioned media from *Gpr35* ^–/–^ M2 macrophages induced less ERK1/2 phosphorylation in H2-11 endothelial cells compared with *Gpr35* ^+/+^ supernatants (figure 6D and online supplemental figure 5C). Similar to VCAM-1 expression, elevated ERK1/2 phosphorylation in H2-11 endothelial cells exposed to *Gpr35* ^+/+^ macrophages was prevented by pretreatment of macrophages with ouabain or pNaKtide (figure 6D). In contrast, endothelial ERK1/2 phosphorylation was not affected when triggered by supernatants of inhibitor-treated *Gpr35* ^–/–^ macrophages (figure 6D). Src and ERK1/2 phosphorylation was correspondingly lower in *Gpr35* ^–/–^ compared with *Gpr35* ^+/+^ tumours (figure 6E and online supplemental figure 5D). This hence demonstrated that GPR35-modulated activation of Na/K-ATPase, and Na/K-ATPase-dependent Src activation, in macrophages controlled Erk1/2 phosphorylation and VCAM-1 expression on endothelial cells via transmissible signals.

### Gpr35 blockade with a GPR35 specific pepducin reduces tumour size in the AOM/DSS-induced CAC model

We have previously shown that treatment of wild-type mice with a GPR35-selective, inhibitory pepducin (g35i2) reduced the number of inflammatory colon tumours.^10^ In the AOM/DSS model, the number of macroscopically visible tumours in g35i2-treated wild-type mice decreased fivefold, whereas the number of microscopic tumours decreased only threefold (figure 6F). Src Tyr^416^ phosphorylation in AOM/DSS tumours was substantially lower in g35i2- compared with control-treated wild-type mice, and was accompanied by reduced Thr^202^/Thr^204^ phosphorylation of ERK1/2 (figure 6G). Furthermore, CXCL-1, VEGF, MMP2 and MMP9 levels as well as immune cell infiltration were significantly decreased when wildtype mice were treated with the g35i2 compound (figure 6H). Altogether this demonstrated that pharmacological GPR35 blockade reduced tumour size, involving the Na/K-ATPase—Src pathway delineated herein.

## Discussion

Here, we report that GPR35 on macrophages is a major promoter of tumour growth by facilitating tumour neovascularisation. We show that this activity is dependent on Na/K-ATPase, and Na/K-ATPase-dependent activation of the non-receptor tyrosine kinase Src within macrophages. The profound reduction in tumour growth observed in both spontaneous and inflammation-induced intestinal tumour models on macrophage-selective deletion of *Gpr35* identifies a novel major mechanism, and vividly highlights the critical role of myeloid cells in tumour development.^18^ We had previously reported that intestinal epithelial cell-specific deletion of *Gpr35* reduces intestinal tumour incidence and reduces epithelial cell proliferation.^10^ Our discovery here adds a major separate tumour-promoting mechanism of GPR35 that originates from TAMs and neutrophils. The UC/PSC-linked T108M polymorphism of GPR35, which is hypermorphic and increases Na/K-ATPase pump activity,^10^ profoundly increases the angiogenic mechanisms as we experimentally demonstrate in human macrophages differentiated from gene-edited iPSCs.^8^ UC is another strong risk factor for the development of CRC.^49^ PSC is the autoimmune disease with the highest lifetime risk of developing cancer,^7^ primarily cholangiocellular carcinoma but also CRC.^7^ GPR35 expression has also been linked to gastric cancer,^50^ and its increased expression in tumour tissue has been strongly associated with poor prognosis in colon cancer and non-small cell lung cancer.^51 52^

Neoangiogenesis is imperative for tumour growth and progression. This is therapeutically exploited by VEGF neutralisation (eg, bevacizumab in CRC^53^), and by IL-8/CXCR2 blockade that is currently investigated in clinical trials.^54 55^ The TME, and TAMs in particular, play an eminent role in tumour angiogenesis.^18^ TAMs appear to derive from circulating inflammatory monocytes that transmigrate into tumour tissue.^56^ TAMs with a predominantly ‘M2-like’ phenotype stimulate angiogenesis by releasing angiogenic cytokines such as VEGF and IL-8.^18 27^ GPR35 deletion does not affect differentiation into the paradigmatic ‘M1’ and ‘M2’ macrophage phenotypes in vitro.^10^ Tumours in mice with macrophage-specific or germline *Gpr35* deletion are smaller, contain fewer CD31^+^ endothelial cells and CD206^+^ M2-like TAMs, and exhibit lower expression of VEGF and CXCL-1, altogether implying reduced angiogenesis as a major component of reduced tumour growth when GPR35 is absent. Enhanced monocyte endothelial transmigration in vitro, and aortic sprout formation ex vivo, further corroborate the critical role of macrophage GPR35 in coordinating an angiogenic response. MMPs, in particular MMP-2, MMP-9 and MMP-14,^57^ facilitate tissue remodelling, a vital process during neo-angiogenesis and tumour progression. MMPs are mostly produced by TAMs, released into the extracellular space as inactive zymogen, and subsequently cleaved and activated by other proteinases.^29^ MMP-9, for example, directly releases glycocalyx-bound VEGF from cell membranes and decreases the amount of syndecan-4 which binds and inactivates VEGF.^58^ The substantially reduced MMP activity in *Gpr35* ^–/–^ tumours corroborates a role of this GPCR as an upstream regulator of tumour tissue remodelling.

GPR35 forms a complex with Na/K-ATPase and promotes its pumping activity, independent of pharmacological or candidate physiological ligands.^10^ Since Na/K-ATPase ascertains electrophysiologic homoeostasis in a cell, reduced pumping activity in GPR35’s absence increases baseline intracellular Ca^2+^, which is maintained via secondary-active transport, to levels otherwise only observed on receptor-mediated signalling events.^10^ Elevated Ca^2+^ in turn affects Ca^2+^-dependent signalling processes broadly.^10 59^ Intriguingly, our results infer a critical role of macrophage Na/K-ATPase pumping activity in (neo)angiogenesis. Teleologically, increasing nutrient and oxygen supply via neo-angiogenesis via this mechanism fits with the notion that maintaining the electrochemical gradient is highly energy-intensive. Increased angiogenic activity of GPR35-sufficient myeloid cells is further dependent on Na/K-ATPase-dependent Src phosphorylation. Cardiotonic steroids such as ouabain block Na/K-ATPase pump function by keeping the pump in its E2P state,^60^ and this pharmacological inhibition of pumping activity had been linked to increased Src phosphorylation.^43^ In contrast, in macrophages expressing GPR35, pNaKtide-inhibitable and hence Na/K-ATPase-dependent Src activation is linked to increased pumping activity.^10^ This relationship of increased Na/K-ATPase activity with increased Src activation is also evident in the aortic sprout assays we report here. This posits interesting questions on the structural basis of GPR35-dependent promotion of pumping activity and Src activation.

In summary, we identify GPR35 on myeloid cells as upstream coordinator of tumour tissue remodelling, reinforcing the critical role of TAMs and neutrophils in cancer progression. The GPR35–Na/K-ATPase–Src pathway is precisely targetable with GPR35-selective pepducins.^10^ Its upstream role in tumour tissue remodelling renders GPR35 an attractive target, even in cases where tumour cells themselves do not express the receptor.

## Data Availability

Data sharing not applicable as no datasets generated and/or analysed for this study. All data relevant to the study are included in the article or uploaded as online supplemental information. n/a.
