# Supplementary material for: Activation of the GPR35 pathway drives angiogenesis in the tumour microenvironment
Source: Gut. 2021 Mar 23;71(3):509–20. doi: 10.1136/gutjnl-2020-323363 (PMC8862021; doi:10.1136/gutjnl-2020-323363)
Supplement: Supplementary data [file gutjnl-2020-323363supp001.pdf]

MATERIALS AND METHODS

Mice

All mice were genotyped using ear biopsy genomic DNA. Animals were bred and housed in specific pathogen free rooms at the Central Biomedical Services facility, University of Cambridge. All procedures were in accordance with the ethical standards of the institution and all were conducted with approval of the U.K. home office. *Gpr35*$^{-/-}$ mice were obtained from the Knockout Mouse Project repository (clone ID 646854). Conditional knockout mice were obtained from the Wellcome Trust Sanger Institute Hinxton (clone ID: 4938582) and then bred into LysMCre transgene–positive mice (Jackson Laboratory, ID: J:67924). Mice developed normally without any spontaneous disease emerging under these breeding conditions. Six- to 12-week-old mice age- and sex-matched mice were used for all experiments unless otherwise stated.

Apc^{min} mice were obtained from the Jackson Laboratory and crossed with mice lacking Gpr35 globally or conditionally in their LysM^{+} cells (i.e. *Gpr35*$^{fl/fl}$;*Apc*$^{min/+}$ and *Gpr35*$^{\Delta M\Phi}$;*Apc*$^{min/+}$).

Primary cell cultures

Bone marrow–derived macrophages (BMDM) were isolated and polarised by standard methods.(10) Macrophages were polarised into M1 and M2 macrophages by adding IFN-γ (50 ng/ml) plus LPS (20 ng/ml) for M1 or with IL-4 (20 ng/ml) for M2, respectively. Murine CD31^{+} endothelial cells were isolated from heart, lung, liver and spleen of 6-8 week-old mice. Organs were incubated in type I collagenase 2mg/ml in PBS (supplemented with $CaCl_2$ and $MgCl_2$ and BSA 1%) at 37°C for 1h. Cells were then incubated with CD45 MicroBeads (Miltenyi Biotech) and CD45^{+} cells were depleted. Unlabelled cells were collected and magnetically separated with antiCD31-MicroBeads (Miltenyi Biotech). Human neutrophils were isolated from peripheral blood of healthy volunteers by standard techniques. In short, blood was separated by Ficoll-Hypaque (Lymphoprep, StemCell Technologies) density gradient centrifugation, after sedimentation red blood cells were lysed and RNA was isolated. For human macrophages peripheral blood was separated by density gradient centrifugation (Lymphoprep, StemCell Technologies), mononuclear cell

Pagano E, *et al. Gut* 2022; 71:509–520. doi: 10.1136/gutjnl-2020-323363

layers were collected, washed and plated in RPMI/10% FBS containing 100ng/mL of GM-CSF (Miltenyi Biotech). RNA was extracted after 6 days in culture.

Cell lines

Murine endothelial cells (H2-11, SVEC) and human endothelial cells (EA.hy926, HUVEC) were purchased from ATCC. Cells were maintained in Endothelial Cell Growth Medium 2 (ECGM2, PromoCell, Heidelberg) at 37°C in 5% CO_2.

CRISPR/Cas9 editing of human iPSC line

The rs3749171 mutation in the human *GPR35* gene was generated by a single base substitution (C>T) using CRISPR/Cas9-induced homology directed repair in the Kolf2 human iPSC line as previously reported. (10)

iPSC culture and macrophage differentiation

KOLF-2 human induced pluripotent stem cells were maintained in mTeSR-E8 medium (Stemcell Technologies) on Vitronectin (rhVTN-N) coated plates (Gibco #A14700). These cells were then differentiated to monocytes using the STEMdiff Monocyte Kit (StemCell Technologies) as per the manufacturer’s instructions. After 14 days, macrophage precursors were harvested from the supernatant and plated on standard tissue culture plates in RPMI 1640, 10% FBS and 1% L-glutamine supplemented with 100 ng/mL M-CSF. After 7 days, mature macrophages were used for assays.

AOM/DSS model of colitis associated cancer

Six- to 8-week-old mice were injected intraperitoneally with AOM (12.5 mg/kg) (Sigma-Aldrich). Colitis was induced by two cycles of 2.5% DSS (MP Biomedicals) in drinking water for 5 days, followed by a 16-day tap water period. The final DSS cycle (2%) was administered for 4 days, followed by a 10-day tap water period. Tumour count and tumour area were determined microscopically (2-4× magnification) after 61 days. Tissues were paraffin embedded or frozen for further analysis.

Pagano E, *et al. Gut* 2022; 71:509–520. doi: 10.1136/gutjnl-2020-323363

Transendothelial migration

H2-11 cells were grown to confluence on 5-µm–pore size 24-well transwell inserts, which separated the upper and the lower chambers. Bone marrow derived monocytes, stained with calcein-AM were resuspended in RPMI 1640, 0.5% bovine serum and migrated towards macrophage conditioned media for 4h. Fluorescence intensity was then measured in the lower wells, and after washing on the transwell inserts to determine adhesion.

Tube formation assay

48 well plates were coated with 100 µl Geltrex (Thermo Fisher) at 4°C, incubated in a humidified incubator (37°C, 5% CO_2) for 30 min. 20,000 freshly passaged calcein-AM stained H2-11 or SVEC cells (passage 2-5 were plated on Geltrex-coated plates. Supernatants from BMDM or iPSC/derived macrophages were added to the endothelial cells and endothelial tube morphogenesis was carried out in the presence or absence of ouabain (100µM) or pNaKtide (1µM). Endothelial tube formation was observed after 6 h and endothelial tubes were photographed. Quantification of tube formation was performed in a blinded manner by counting total tubes per five 40x fields and quantified for tube length and branch complexity using NIH ImageJ software.

Aortic ring assay

Mice were euthanised and aortas collected. The connective tissue was then removed and aortas cut into 1-2mm rings which were left in Opti-MEM overnight. On day 1 of the experiment rings were placed into collagen-coated 96 well tissue plates and Opti-MEM containing 2.5% FBS and 30ng/mL VEGF was added. Sprouts were counted blindly on a daily basis. For some experiments, kynurenic acid (KYNA) (100 µM, Sigma Aldrich), CXCL-17 (20 ng/mL, BioLegend), ouabain (100µM, Sigma Aldrich), pNaKtide (1 µM, peptide 2.0), polyclonal anti-VEGF antibody (100 ng/mL, Invitrogen) or the CXCR2 blocking pepducin x1/2pal-i3 (3 µM, peptide 2.0) were added on day 3. Total number of rings on day 9 is shown.

Pagano E, *et al. Gut* 2022; 71:509–520. doi: 10.1136/gutjnl-2020-323363

ELISA assays

CXCL-1, VEGF, MMP-2, MMP-9 and IL-1β from mouse tumour tissue or macrophage supernatants were measured by standard ELISA technique. Quantikine ELISA assays were bought from R&D systems and cytokines assessed adhering to the protocols.

MPO assays

Tumour tissue was homogenised and MPO was measured and calculated adhering to the manufacturer's protocol (Sigma Aldrich).

Western blotting

Cells or tissue were lysed in RIPA buffer and equal amounts of lysates were separated by SDS–polyacrylamide gel electrophoresis. After blotting onto Hybond P membranes (GE Healthcare), blots were blocked with 5% milk, and primary antibody was added at 4°C overnight. The protein was then detected by using an HRP-conjugated secondary antibody and visualized with LumiGLO (Cell Signaling Technology).

Immunofluorescence

Frozen sections (5 μm) of murine colon tissue from AOM/DSS model were fixed with 100% acetone at -20°C for 20 minutes and then rinsed with PBS containing 0.05% Tween (PBS-T). After blocking, sections were incubated overnight at 4°C with rabbit anti-CD31 (1:50; BD Bioscience) and goat anti-CD206 (5μg/mL; Invitrogen) primary antibodies. Sections were subsequently incubated for 30 minutes with either anti-rabbit Alexa Fluor 488− or anti-goat Alexa Fluor 650 (1:100; Abcam). Coverslips were mounted with ProLong mounting media containing DAPI (Invitrogen) and fluorescence was analysed with a Leica SP5 confocal microscope.

Pagano E, *et al. Gut* 2022; 71:509–520. doi: 10.1136/gutjnl-2020-323363

Immunohistochemistry

Paraffin sections were pre-treated with xylene and dehydrated in ethanol. Antigen retrieval was performed using citrate buffer for 15 min at sub-boiling temperature in a microwave, followed by blocking of endogenous peroxidases activity. Sections were incubated with the anti-VCAM antibody (D2T4N, Cell Signaling Technology) overnight at 4°C and detected with HRP SignalStain® Boost IHC Detection (Cell Signaling Technology). Sections were developed using a DAB Peroxidase Substrate kit (Cell Signaling Technology).

FACS analysis of tumours

AOM/DSS tumours were collected and digested using the murine tumour dissociation kit (Miltenyi, 130-096-730). Single cell suspensions were blocked with anti-mouse CD16/32 (Biolegend, 101301), and stained with antibodies against CD31 (390), CD68 (FA-11), CD206 (C068C2), CD11c (N418), MHCII (M5/114.15.2), CD163 (S15049I), Ly-6G (1A8) (all purchased from Biolegend). Viability was assessed by staining with SYTOX™ Blue Nucleic Acid Stain (ThermoFisher). Samples were measured on Attune NxT flow cytometer and analysis performed using FlowJo v10. An example for the gating strategy is given in Supplementary Figure 2D.

Gelatine zymography

Cell lysates or supernatants were resolved in 12% polyacrylamide gels containing (1 mg/ml) gelatine A (Sigma). After electrophoresis, gels were washed three times in in regeneration buffer (20 min) followed by incubation in developing buffer (18 h at 37°C) and stained with 0.25% Coomassie blue and then destained.

RNA extraction and reverse transcription quantitative polymerase chain reaction

RNA was isolated using the RNeasy Mini Kit (QIAGEN). RNA was then reverse-transcribed and SYBR Green (Eurogentec) quantitative polymerase chain reaction was performed using Mx3000 (Agilent Technologies). Target gene expression is expressed as ratio to housekeeping gene expression.

Pagano E, *et al. Gut* 2022; 71:509–520. doi: 10.1136/gutjnl-2020-323363

Statistical analysis

Statistical analysis was performed using Prism 8 (GraphPad) software. The statistical tests used in each experiment are described in the respective figure legends.

Supplementary table 1.

REAGENT or RESOURCE	SOURCE	IDENTIFIER
Antibodies		
pSrc	Cell Signaling Technology	D49G4
Src	Cell Signaling Technology	36D10
pErk	Cell Signaling Technology	9101
Erk	Cell Signaling Technology	137F5
VCAM	Cell Signaling Technology	D2T4N
pVEGF receptor 2	Cell Signaling Technology	19A10
VEGF receptor 2	Cell Signaling Technology	55B11
b-actin	Cell Signaling Technology	13E5
VEGF	Invitrogen	PA5-47021
CD31	BD Bioscience	550274
CD206	Invitrogen	PA5-46994
Chemicals, Peptides, and Recombinant Proteins		
pNaktide	Peptide 2.0	
x1/2pal-i3	Peptide 2.0	
g35i2	Peptide 2.0	
Ouabain	Sigma-Aldrich	Cat# O3125
Kynurenic acid (KYNA)	Sigma-Aldrich	Cat# K3375
CXCL-17	BioLegend UK	Cat# 585404
Dextran sulfate sodium salt, colitis grade (36,000 - 50,000)	MP Biomedicals	160110
Geltrex	Thermo Fisher	A1413201
Azoxymethane	Sigma-Aldrich	A5486
Critical Commercial Assays		
Mouse IL-1 beta/IL-1F2 Quantikine ELISA kit	R&D Systems	MLB00C
Mouse CXCL-1/KC Quantikine ELISA kit	R&D Systems	MKC00B
Mouse VEGF Quantikine ELISA kit	R&D Systems	MMV00
Experimental Models: Cell Lines		
2H11	ATCC	CRL-2163
SVEC	ATCC	CRL-2181
HUVEC	Promo-Cell	C-12206
EA.hy926	ATCC	CRL-2922
HMVEC	Promo-Cell	C-12212
Experimental Models: Organisms/Strains		

Pagano E, *et al. Gut* 2022; 71:509–520. doi: 10.1136/gutjnl-2020-323363

Gpr35$^{fl/fl}$ mice	Wellcome Trust Sanger Institute	ID: 4938582
Gpr35$^{-/-}$	Knockout Mouse Project	ID: 646854
LysMCre transgene mice	Jackson Laboratory	ID: J:67924
APC^{min} mice	Jackson Laboratory	Cat# JAX:002020, RRID:IMSR_JAX:00 2020
Oligonucleotides		
Human betaActin Qrev GGGGTACTTCAGGGTGAGGA	Sigma-Aldrich	
Human betaActin Qfwd GATGCCCCCCGGGCCGTCTT	Sigma-Aldrich	
Murine Beta Actin forward GCCCTGAGGCTCTTTTCCAG	Sigma-Aldrich	
Murine Beta Actin reverse TGCCACAGGATTCCATACCC	Sigma-Aldrich	
Murine gpr35_fwd AAATCCCCACCTTTCAGCACA	Sigma-Aldrich	
Murine gpr35_rev CATGGTCCTAGGGCTCATCTG	Sigma-Aldrich	
Human gpr35_fwd GCTCACCCCAGCTTCACTTC	Sigma-Aldrich	
Human pr35_rev TAGGTGCCATTCATGGTCCTGC	Sigma-Aldrich	
Software and Algorithms		
Graph Pad Prism 8	Graph Pad Software Ltd	

Pagano E, *et al. Gut* 2022; 71:509–520. doi: 10.1136/gutjnl-2020-323363

Supplementary Figure 1. GPR35 does not affect the course of acute DSS induced colitis. Weight changes in acute DSS colitis. **A.** *Gpr35*$^{+/+}$ and *Gpr35*$^{-/-}$ mice subjected to 3.5% DSS in their drinking water for 5 days, weight monitored daily for 7 days. N = 8/group. **B.** *Gpr35*$^{fl/fl}$ and *Gpr35*$^{\Delta M\Phi}$ mice subjected to 3.5% DSS in their drinking water for 5 days. Weight was monitored daily for 7 days. N = 7/group. **C.** Weight changes in the AOM/DSS model. After an initial injection of AOM, mice were subjected to 3 cycles of 2.5% DSS on their drinking water (arrows). N = 15 mice, whereby 4 mice needed to be euthanised in the *Gpr35*$^{fl/fl}$ group and 1 mouse in the *Gpr35*$^{\Delta M\Phi}$ group as they reached the humane weight loss limit. Statistical significance was calculated using multiple t-tests. **D.** Survival rate in *Gpr35*$^{fl/fl}$ and *Gpr35*$^{\Delta M\Phi}$ mice for AOM/DSS induced intestinal tumours. N = 15. Statistical significance was calculated using the Mantel-Cox test. **E.** Histological assessment of tumour infiltrating immune cells in the AOM/DSS model. N = 10 for *Gpr35*$^{fl/fl}$ and N =. 12 for *Gpr35*$^{\Delta M\Phi}$. **F.** MPO activity in *Gpr35*$^{fl/fl}$ and *Gpr35*$^{\Delta M\Phi}$ tumour tissue (left panel) and in tumours from *Gpr35*$^{+/+}$ *or Gpr35*$^{-/-}$ mice (right panel). Data represented as mean ± s.e.m. Statistical significance was calculated using Mann Whitney *U* after Kruskal Wallis testing.

Supplementary Figure 2. Endothelial cells do not express *GPR35*/*Gpr35* mRNA.
A. Relative mRNA levels of murine primary CD31+ endothelial, murine SVEC and 2H-11 endothelial cells and human HUVEC and HMVEC cells. **B.** *Gpr35* mRNA levels in bone marrow-derived neutrophils and macrophages from *Gpr35*$^{fl/fl}$ and *Gpr35*$^{\Delta M\Phi}$ mice. **C.** *GPR35* mRNA levels in human peripheral blood neutrophils and macrophages. mRNA levels were measured via qRT-PCR and normalized to ACTB (β actin). N = 3 to 4 per group. All data represented as mean ± s.e.m. Statistical significance was calculated using Mann Whitney *U* after Kruskal Wallis testing. **D.** Gating strategy for flow cytometry analysis of tumour tissue. Viability was assessed by staining with SYTOX™ Blue Nucleic Acid Stain. Samples were measured on Attune NxT flow cytometer and analysis performed using FlowJo v10.

Pagano E, *et al. Gut* 2022; 71:509–520. doi: 10.1136/gutjnl-2020-323363

Supplementary Figure 3. *Gpr35*$^{+/+}$ macrophage supernatants stimulate tube formation in 2H-11. 2H-11 cells seeded in Geltrex and incubated with supernatants from *Gpr35*$^{+/+}$ or *Gpr35*$^{–/–}$ macrophages. N = 14 per group. Statistical significance was calculated using Mann Whitney *U* after Kruskal Wallis testing

Supplementary Figure 4. Ouabain does not increase Src phosphorylation in the presence of GPR35. M0 macrophages were probed for Src phosphorylation in the presence or absence of ouabain (100µM). N = 3 per phenotype

Supplementary Figure 5. Density plots of Western blots. Density of all Western blots performed analysed using Image J's Analyzer tool. N = 6 per group. Statistical significance was calculated using Mann Whitney *U* after Kruskal Wallis testing.

Pagano E, *et al. Gut* 2022; 71:509–520. doi: 10.1136/gutjnl-2020-323363
