# Supplementary figures and images for: Activation of the GPR35 pathway drives angiogenesis in the tumour microenvironment
Source: Gut. 2021 Mar 23;71(3):509–20. doi: 10.1136/gutjnl-2020-323363 (PMC8862021; doi:10.1136/gutjnl-2020-323363)

Suppl Fig 1

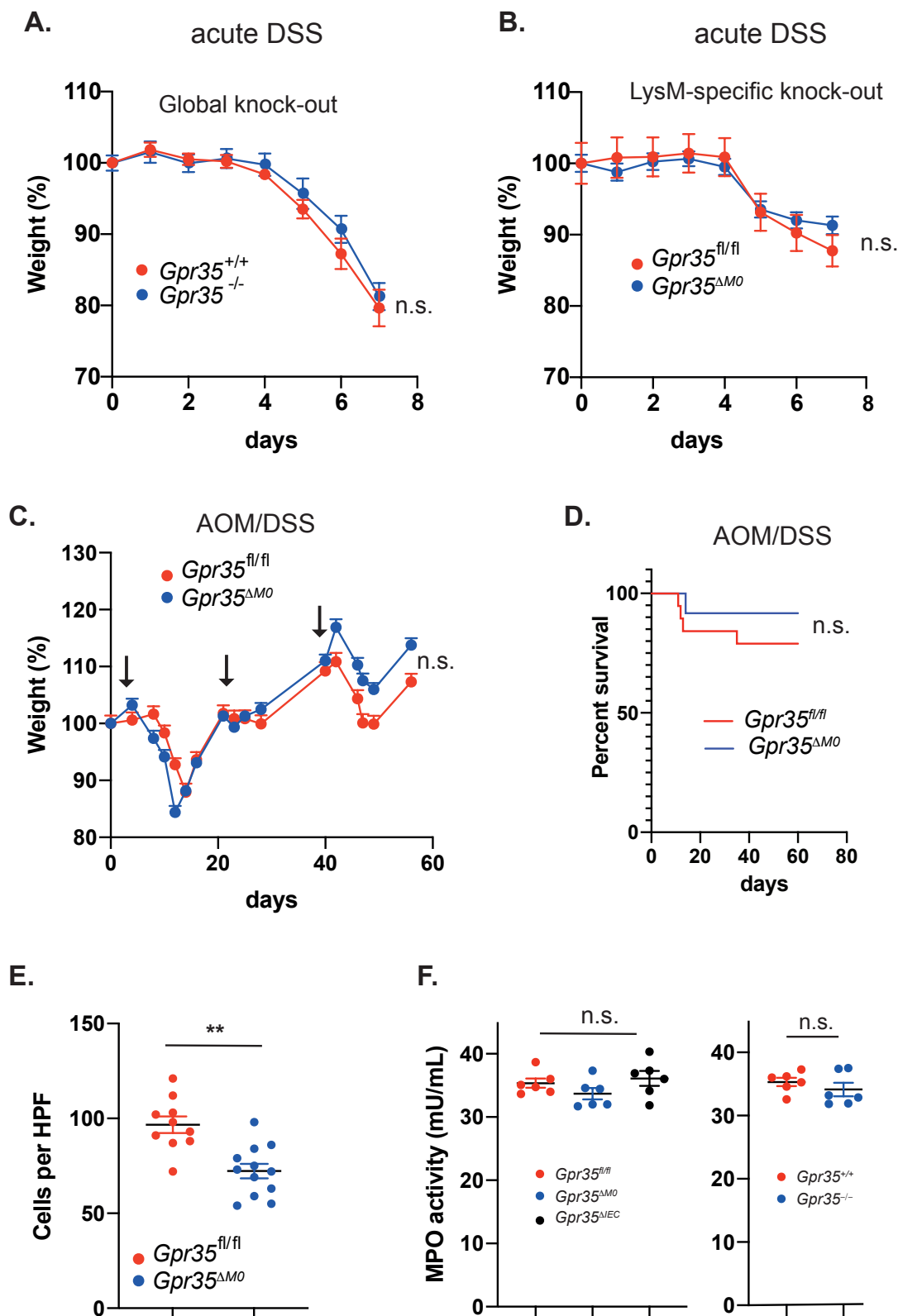

Suppl Fig 1

A.

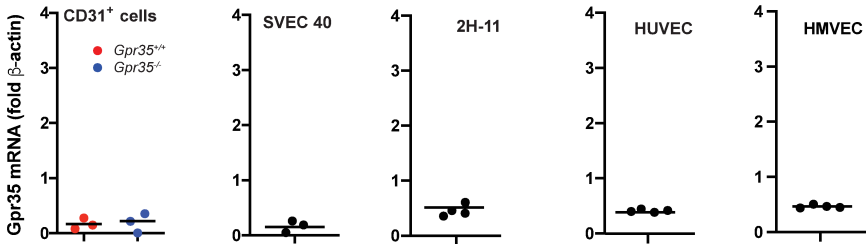

B.

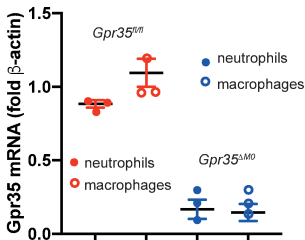

C.

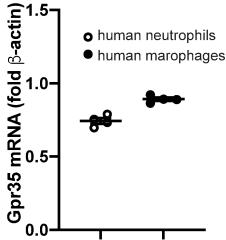

D.

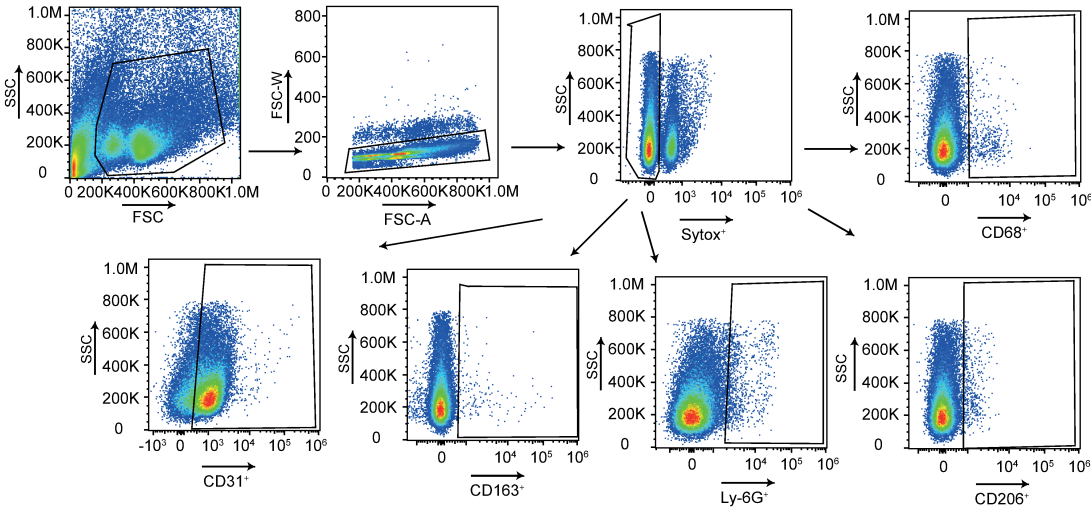

Suppl. Figure 3

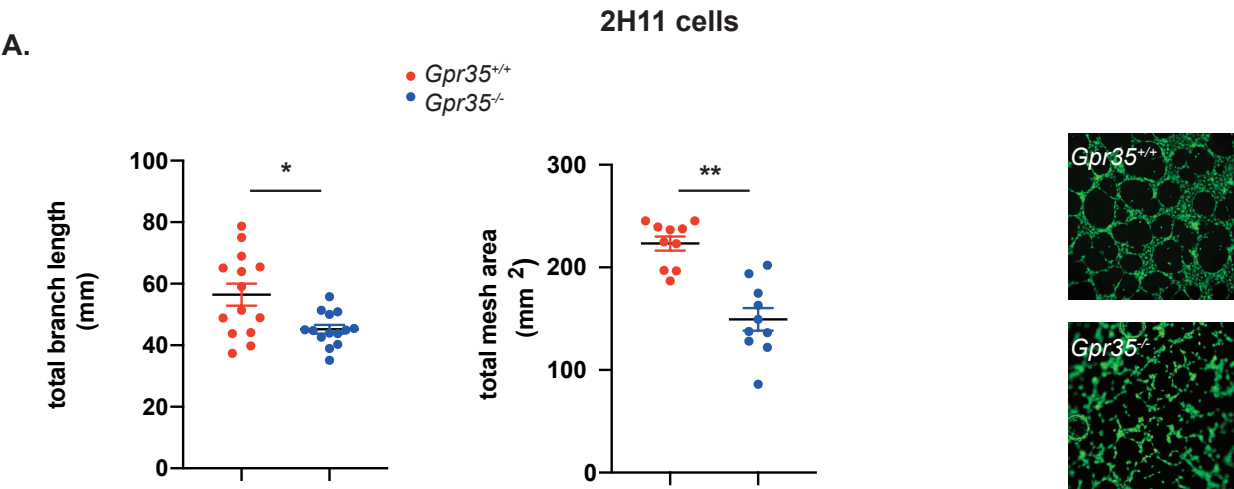

## Suppl Figure 5

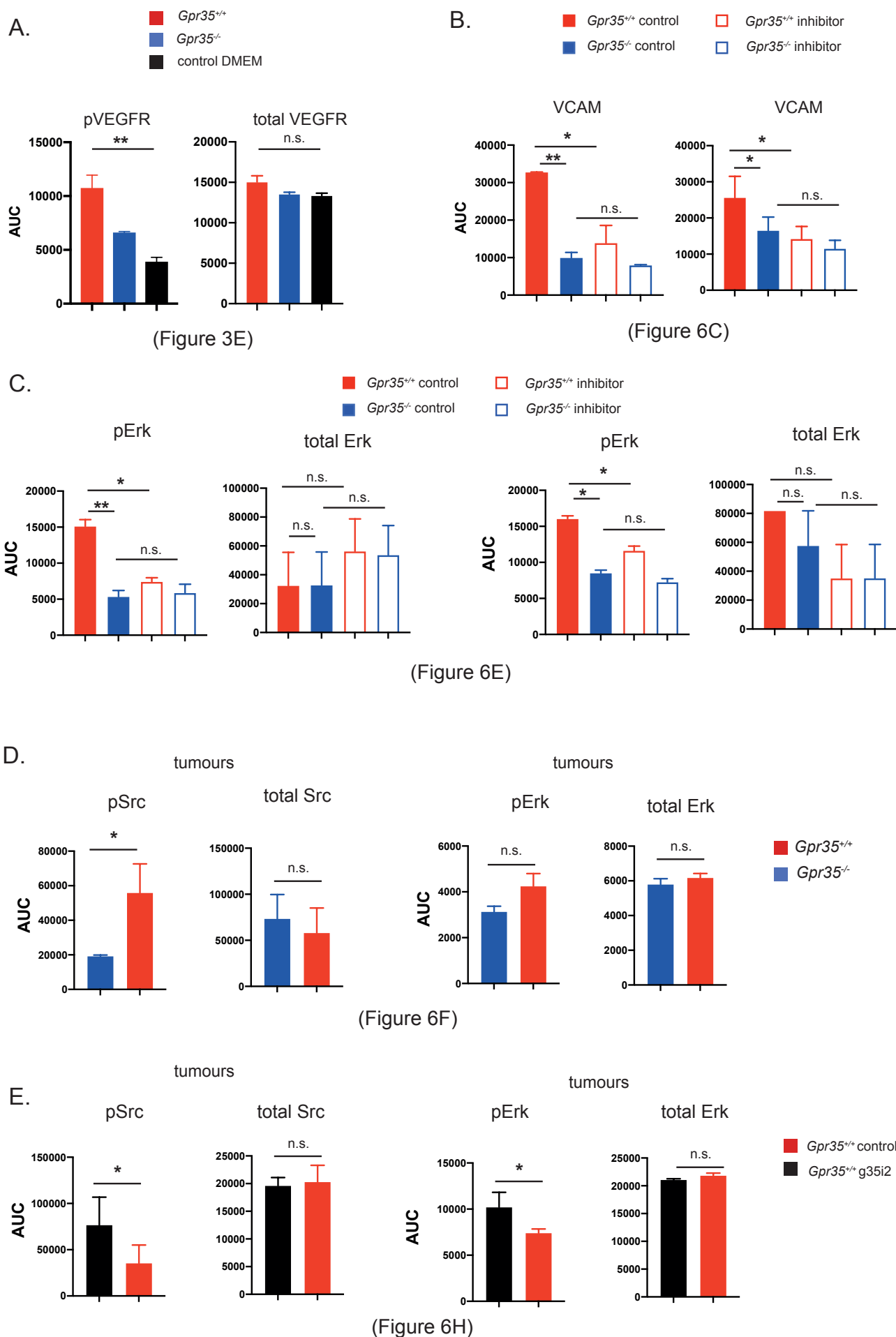

Suppl. Figure 4

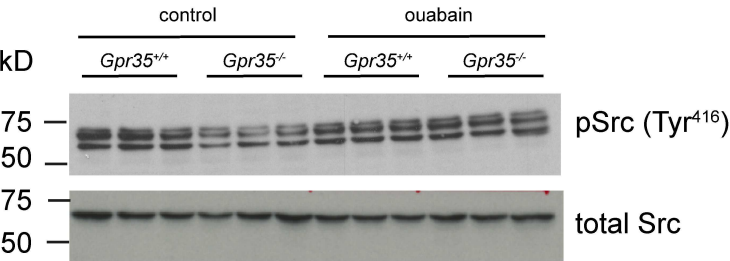

Supplement: Supplementary data [file gutjnl-2020-323363supp002.pdf]
